# Supplementary material for: Global surgery for medical students – is it meaningful? A mixed-method study
Source: PLoS One. 2021 Oct 7;16(10):e0257297. doi: 10.1371/journal.pone.0257297 (PMC8496788; doi:10.1371/journal.pone.0257297)
Supplement: S1 Appendix — (DOCX) [file pone.0257297.s001.docx]

## The Global surgery course At Karolinska Institutet

## Curriculum

The course has two modules: a theoretical part and an abroad clinical elective.

The theoretical part, taking place in Sweden, includes four evening lessons. They highlight global surgery from several angles; ethics, epidemiology, surgical indicators of health, research, development and possibilities and challenges of surgery with limited resources. A very large focus lies on research and development. The course literature is based on the 2015 Lancet Commission of Global Surgery Report.

The second part of the course consists of a two-week clinical elective in Uganda at the regional referral hospital in Mubende or Soroti and at Mulago National Referral Hospital in the capital city, Kampala. These hospitals have been selected to give a direct and real understanding of surgical care and traditions regarding treatment by visiting two different levels of health care facilities. Students participate in ward rounds and general ward duties together with the local nurses and surgeons. All students participate in rotations in general surgery and obstetrics with additional rotations that vary from year-to-year and include surgical oncology, cardiothoracic surgery, burns and plastic surgery, paediatric surgery and orthopaedic surgery. In theatre the students are allowed to observe and often assist during the procedures. A minimum of two teachers from Sweden accompany the students during their rotations in the regional referral hospital and four teachers in the national referral hospital. The Swedish teachers are available during evenings and the weekend for informal reflection and discussions. Two scheduled reflective sessions are held, one per week. The teachers’ role is to guide the students and make sure they get the most out of each rotation, teaching and explaining. The teachers are also in charge of organizational matters to limit the burden of work to the Ugandan partners.

Learning outcomes are divided into three domains: ‘knowledge and understanding’, ‘skills’ and ‘attitude’, see Table 1.

Table 1. Learning outcomes of the global surgery course at KI.

| After the course students should be able to: | | |
| --- | --- | --- |
| Knowledge and understanding | describe and explain | similarities and variations in surgical and obstetric panoramas of disease in low- and middle-income countries and over time; also, how surgical procedures can reduce morbidity and mortality in different contexts, |
|  | compare and analyse | different factors that affect access to surgery such as variations in accessibility and quality of surgical care between and within countries, |
|  | reflect on | differences in the need for surgical care in different settings and the increased need following different catastrophes, |
|  | understand | the "unmet need of surgical care and universal health coverage", describe the Millennial Development Goals and discuss how countries and the Lancet commission on global surgery are trying to reach them, |
| Skills | present | a patient case with a surgical problem and give a reasonable solution to it in the context of a low- and middle-income country, |
| Attitudes | show understanding of | how cultural and economic factors affect patients in different healthcare systems and how to behave in a different culture without abandoning your ethical approach towards patients, |
|  | act | respectfully towards patients, other students, teachers and staff and take responsibility for their own education and professional development. |

By the end of the two weeks the students are assessed on an oral presentation of a patient case they have encountered. Students are instructed to discuss aspects of global surgery, differences between Swedish and Ugandan health care and the theory of the “three-delay framework” (i.e., delay in seeking care, reaching care and receiving care) associated to the case. A formative assessment of theoretical knowledge is carried out by the students at the first course meeting and on the last day in Uganda. See Table 2 for an illustration of the course-structure after implementing changes suggested in this article. The formative assessment and the global surgery introduction are always held at the first course meeting. The order of the other specific lectures varies between the semesters.

Table 2: Example of course structure and content for the Global surgery course at KI

|  | | Educational elements |
| --- | --- | --- |
| Sweden – theoretical part | Lesson 1 | Formative assessment.  Lectures:  Global surgery – an overview.  Ethics and international rotations.  Case-based ethics exercise.  Practical information about the course and the Uganda trip. |
|  | Lesson 2 | - Lecture:   - Research and global surgery. - Journal club where the students discuss and present a preselected publication in small groups. |
|  | Lesson 3 | - Lectures:   - Anaesthesia and work in international organisations.   - Oncology in a global perspective. |
|  | Lesson 4 | - Lectures:   - Gynaecology and obstetrics. |
|  | Informal meeting | - Previous students present their experiences from the course. - Presentation of global surgery master thesis. |
| Uganda – clinical elective | Week 1 – regional referral hospital | - Rotations in general surgery and gynaecology and obstetrics. - Reflection. |
|  | Week 2 – national referral hospital | - Rotations in thoracic-, paediatric- orthopaedic- and/or plastic surgery and oncology. - Reflection. - Oral examination. - Formative assessment. |
