# Supplementary material for: Global surgery for medical students – is it meaningful? A mixed-method study
Source: PLoS One. 2021 Oct 7;16(10):e0257297. doi: 10.1371/journal.pone.0257297 (PMC8496788; doi:10.1371/journal.pone.0257297)
Supplement: S2 Appendix — (DOCX) [file pone.0257297.s002.docx]

**Formative Assessment**

1. How many people in the world lack access to safe surgery, at a reasonable cost when needed?
   1. 3 billion
   2. 5 billion
   3. 500 million
2. What are the so-called Bellwether Procedures?
   1. caesarean, laparotomy and operative management of open long-bone fractures
   2. caesarean, hernia surgery and mastectomy
   3. wound suturing, drainage of abscesses and male circumcision
3. Which are globally equally common conditions?
   1. Deaths due to malaria, tuberculosis and HIV/AIDS infections compared to deaths due to surgical diseases
   2. Inguinal hernias and conditions due to female genital mutilation
   3. Appendicitis and clubfoot
4. How great is the economic loss due to untreated surgical conditions in relation to the cost to perform the needed surgery?
   1. 12 billion kronor/year vs 30 billion kronor/year
   2. 12 trillion dollars vs 300 billion dollars for the years 2015-2030
   3. They are approximately the same – to give access to surgical care has mainly ethical implications
5. What is the difference of task-sharing and task-shifting?
   1. No difference
   2. Task-shifting: a person performs task they are not educated in. Task-sharing: a person receives education to perform assigned tasks.
   3. Task-shifting: re‐distribution of tasks from highly qualified health workers to health workers who have fewer qualifications without highly qualified assistance. Task-sharing: tasks are shared in teams where workers with fewer qualifications perform tasks with access to help from highly qualified personnel.
6. Access to health care is unevenly distributed in the world, what percentage of global surgical care is performed in the poorest 1/3 of the world?
   1. 1%
   2. 3,5%
   3. 6%
7. The surgical frequency is recommended to be 5000 procedures/100 000 people per year to meet the basic surgical need in a population. That equals a global lack of 143 billion procedures/year. In what ball-park is the surgical frequency in many low-income-countries?
   1. 3000 / 100 000 per year
   2. 300 / 100 000 per year
   3. 3 / 100 000 per year
8. Describe the principles of the “three-delays framework”:
9. How can research and a scientific approach contribute to improve the situation people with surgical conditions in low- and middle-income countries?
10. State and describe three innovations that could improve surgical care in low- and middle-income countries:
