# Supplementary material for: Global surgery for medical students – is it meaningful? A mixed-method study
Source: PLoS One. 2021 Oct 7;16(10):e0257297. doi: 10.1371/journal.pone.0257297 (PMC8496788; doi:10.1371/journal.pone.0257297)
Supplement: S3 Appendix — (DOCX) [file pone.0257297.s003.docx]

Case based interviews

Translated from Swedish 2021-04-15 by the first author (SK).

**Background information**

Thank you for participating in this study, the aim of the study is to improve the global surgery course at KI and explore its impact on participating students.

All final results from this study will be anonymized when published.

Please start by answering the following demographic questions:

Name:

Gender:

- Female
- Male
- Other
- Prefer not to say

Age: ___

Number of full-time semesters at university: _________

The semester you participated/will participate in the global surgery course: ______

I have been on a 2 week elective in Uganda with the global surgery course at KI:

- Yes
- No

I have previous experience of electives abroad in a healthcare setting:

- Yes
- No

If yes, where and for how long? _________________________________________________

I have had previous participation in a similar course:

- Yes
- No

If yes, where and what type of course? ___________________________________________

How do you feel about future engagement in global surgery on any level, for example through research or clinical work abroad? _________________________________________________________________________________________________________________________________________________________________________________________________________________________________

# Case 1 – A feverish three-year-old

**Your role:** You were lucky enough to get an internship right after your medical school graduation. You are currently working as an intern doctor at a hospital, and During you medical rotations you have a two month placement in pediatrics. Today you are on call at the pediatric emergency department (ED).

**The patient:** Esther is a three years old presenting to the ED with her mother. She has had an fever since this morning, and her mother has brought her to the hospital for treatment. The nurse looking after the little girl approaches you, and in an annoyed tone asks: “Why do they always find the need to come to ED?”

On examination, you find that Esther has a fever of 38,5°C, but nothing else of concern. she is alert, playful and lets you examine her without fuss.

1. **Anything else you wish to know about the patient?** *If the student asks more questions to the parents give answers as presented below:*
   - **Recent travel to a malaria endemic area?** Have not been abroad in the past year.
   - **Social history?** Born and raised in Sweden, parents are from Sub-Saharan Africa, used to live in one of the Migration Agency’s accommodation centers but now lives in an apartment. She is the youngest of 3 siblings.
   - **Past medical history?** There is nothing else of relevance in her history.

You conclude that Esther likely has a form of viral infection, but is clinically well. You recommend they give paracetamol to lower the fever and urge them to come back if she gets worse or does not improve in the next few days. You also advise that should a similar scenario arise in the future, primary care should be their first port of call.

Esther’s mother does not react quite the way you anticipated. You feel that she expects a prescription. You get a bit frustrated as you are in a hurry to see the other waiting patients, and it’s common knowledge that you should not prescribe antibiotics for a viral infection. You try to explain this to Esther’s mother, but she still does not seem convinced.

1. **What do you believe the problem is in this situation?**

Esther’s father now enters the room. he is worried about his daughter and ask you what medication you will prescribe. You repeat your conclusions and a verbal conflict arises; he demands that you write a prescription. You do not want to bother you consultant again. Eventually, they agree to come back in 2 days for a follow-up consultation.

**Two days later.**

Today is your scheduled follow-up appointment with Esther and her parents. you are not looking forward to it. Its uncomfortable with stressful patient consultations.

1. **How do you prepare yourself for this appointment?**

You bring the girl and her parents in to your office. You realize Esther is now fully recovered and nothing more needs to be done.

1. **What do you do know?**

*If the students decide to speak with the parents further there is more information below to be given as answers to specific questions. If they do not ask more questions give this information as an epilogue.*

Ether’s parents came to Sweden with her siblings shortly before she was born. The father worked as a teacher and the mother was forced to quit school in 5^th^ grade after she became pregnant with their first child. Their first child died when he was 4 years old, and the mother was home alone with him when he got a high fever. She waited the whole day for the father to get back from work to have money to go to the hospital. She got paracetamol and some herbs from a neighbor and when they finally accessed the community health center, they were given anti-malaria drugs but, unfortunately, he did not improve in the following days. After a few days they reached the regional hospital; however, upon arrival the doctors said it was too late. They were able to buy a cannula and a bag of fluids. They were told they should have come earlier as the boy was now very sick with pneumonia and he could no longer be saved. Unfortunately, he died soon after.

**Questions after the case:**

1. **What are your feelings in relation to this case?**
   - During the emergency department visit?
   - During the scheduled appointment two days later?
   - In what way were you affected by the nurse’s comment at the emergency department?
2. **How do you think the patients and parents background contributed to the course of the consultations?**
   - What do you believe the parents were worried about?
   - How did you resonate about differential diagnoses in relation to the patients’ background?
   - What ideas, concerns and expectations do you think the parents had during the two hospital visits?
   - What ideas, concerns and expectations did you have during the two hospital visits?
   - How do you feel in regards to meeting a patient of immigrant background?
   - Do you have any previous experiences you think affect your feelings?
3. **What have you learned from this case that you can make use of in future consultations?**
4. **Is there anything you wish to discuss before moving on to the next case?**

# Case 2 – secondary headache and depression

**Your role:** you have temporary employment at a primary health care facility in Stockholm during the summer. It’s Wednesday afternoon and you have six appointments left: dizziness, fatigue, weight loss, headache, abdominal pain and dizziness again.

You prepare to meet patient number four. you have 20 minutes for the consultation, are already behind on time and really need to get home on time today.

**The patient:** You call in a middle-aged woman from the waiting room and try to start building rapport on the way to your office. It does not go very well as she refuses to make eye-contact. Once seated you start asking the routine questions to differentiate potentially fatal headaches from the benign ones. There are no red flags, and you conclude its likely tensions headache and recommend prescription-free analgesia and physiotherapy. She does not seem to fully grasp this, and you get the feeling there is something else going on that she is not telling you. She seems despondent. Gah! How are you supposed to have time for this as well!?

You schedule a second appointment the following week and have her leave a blood sample on the way out.

1. **Do you do anything else before she leaves?**
2. **What do you believe the problem is in this situation?**

**One week later.**

1. **How do you prepare yourself for this appointment?**

You have the patient before you in your office. She is still avoiding eye-contact.

1. **How do you move forward with this case?**

**Further description of the patient to answer specific questions:**

The patient deems herself of good health. she is not on any medication. She is not pregnant and does not smoke or drink alcohol. She has no risk factors for common non-communicable diseases. She has tried over the counter analgesia but still has a headache and body aches.

On screening she fulfills the criteria for a depressive episode.

The patient was born in Ethiopia. She has been in Sweden for three years and lives with her husband and five children (three girls aged six, eight and nine and two boys aged thirteen and fifteen). Her marriage was arranged. She has a high-school diploma and is currently studying Swedish. During the days she tends to hang out with other Ethiopian women in the area. The sole family income comes from her husband’s small grocery store.

At aged four, she was mutilated resulting in severe suffering with painful menstruations and tears during labor which ultimately made her incontinent. She has not been able to talk to anyone about this. The one time she tried to discuss her struggles, it was dismissed as part of the natural body changes that occur after childbirth.

As her daughters have been getting older, this topic has become more stressful, especially since friends have started talking about mutilating their children. She has been told you can bring them back to Ethiopia to have it done, and she thinks her husband is making such plans for their kids and her mother-in-law’s pushing. They have scheduled a visit to Ethiopia this summer, and the worry for her daughters is the key reason for her headaches and unhappiness.

**Questions after the case:**

1. **What are your feelings in relation to this case?**
   - During the first visit?
   - During the second visit?
2. **How do you think the patients background contributed to the course of the consultations?**
   - What do you believe the parents were worried about?
   - How did you resonate about differential diagnoses in relation to the patients’ Sub-Saharan background?
   - What ideas, concerns and expectations do you think the parents had during the two hospital visits?
   - What ideas, concerns and expectations did you have during the two hospital visits?
   - How do you feel in regards to meeting a patient of immigrant background?
   - Do you have any previous experiences you think affect your feelings?
3. **What have you learned from this case that you can make use of in future consultations?**
4. **Is there anything you wish to discuss before we finish?**
