# Supplementary material for: Global surgery for medical students – is it meaningful? A mixed-method study
Source: PLoS One. 2021 Oct 7;16(10):e0257297. doi: 10.1371/journal.pone.0257297 (PMC8496788; doi:10.1371/journal.pone.0257297)
Supplement: S4 Appendix — (DOCX) [file pone.0257297.s004.docx]

# Complementary results: course evaluation

Of the 47 course participants who were invited to evaluate the course, 38 (80.9 %) responded.

**Table 1: Student course evaluation: students’ ratings of different course elements from the standardized questions.**

| Question | Responses | | | |
| --- | --- | --- | --- | --- |
|  | n | Median [IQR] | Min | Max |
| What is your overall impression of the course? | 38 | 5 [0] | 4 | 5 |
| I would recommend the course to someone else | 38 | 5 [0] | 4 | 5 |
| I believe I will be able to make use of my increased knowledge and understanding from the course in my clinical work as a doctor. | 38 | 5 [0] | 3 | 5 |
| I believe the course stimulated a scientific approach, for example the use of analytical and critical thinking, information seeking and ability to evaluate information. | 38 | 4 [1] | 3 | 5 |
| The course improved my knowledge of health-promoting and disease-preventing interventions | 37* | 5 [0] | 3 | 5 |
| I reached the goals of the course | 38 | 4 [1] | 3 | 5 |
| The curricular aims were clear to me | 38 | 4 [1,25] | 2 | 5 |
| I received valuable feedback on my achievements during the course | 38 | 4 [2] | 2 | 5 |
| The examination was relevant in relation to the curricular aims | 38 | 5 [1] | 3 | 5 |
| The clinical training was relevant to the curricular aims | 37* | 5 [0] | 4 | 5 |

Number of responding students = n, missing data = *. Students responses are presented as median and interquartile range [IQR]. Responses given on a 5-point Liker-scale (1 – strongly disagree, 2 – disagree, 3 – neutral, 4 – agree and 5 – strongly agree or 1 – very bad, 2- bad, 3 – neutral, 4-good, 5 – very good).

### Students opinion about the course

Students appreciated Uganda and the two hospitals as locations for the overseas experience, as they got to see the difference within the country, its resources and also seeing the countryside versus the capital. In general, the students found it easier to be active and participate in clinical work in Mubende. They appreciated being able to communicate with staff and often patients in English. They acknowledged the need to understand Uganda’s political and social situation and requested an introduction on these matters to fully grasp the context.

“Kampala was cool and a big contrast to Mubende. It was educational to see that there is highly specialized care also in Uganda (which did not seem to be the case in Mubende)” Student 11, autumn 2017.

Students appreciated the doctor and nurses in Uganda and their teaching abilities as part of the ‘educational environment’. Major ward rounds were in general considered crowded and hard to follow and smaller group activities were appreciated. They suggested being paired with a member of staff or medical student to maximize the experience. They also appreciated being able to participate during surgery in Mubende. They suggested having a Swedish teacher present as essential to their educational outcome, since the teacher could describe procedures, translate when necessary to Swedish and highlight differences from and similarities to the Swedish setting. They highlighted the course leaders’ personal connections to the country as very beneficial.

“Ward rounds were in general hard to follow/difficult to hear. When it was bad it was a waste of time. When they were good, they were great! Unfortunately, they were more often bad. Maybe you can encourage to shadow a doctor” Student 5, spring 2017.

“The course offers strong experiences and impressions; you need to process and reflect on site. Therefore, it is of great importance that all groups have a teacher accompanying them during clinic so you can ask questions and discuss what you see” Student 9, spring 2017.

Many students requested longer clinical rotations, one reason being that with time they learned how to make most use of the clinical experience.

“The clinical part abroad t should be longer to give greater opportunity to gain knowledge and understand global surgery”. Student 1, spring 2018.

Students expressed a need for more previous preparation, information and communication regarding what to expect and prepare. They were positive towards written summaries and information from previous students. They understood the need to make last-minute changes and that things could run late but wanted a more robust communication strategy to ensure that all get the same information.

Students wanted clearer curricular aims and information about the examination. A majority of those commenting on the lectures and course structure in Sweden appreciated that they gave an overview of the subject, and they requested more resources for self-studies. Some requested more lectures and the addition of other subjects; some wanted more Uganda-specific lectures instead of a global approach. A few students wrote that the evening lectures were too long.
