# Supplementary material for: Global surgery for medical students – is it meaningful? A mixed-method study
Source: PLoS One. 2021 Oct 7;16(10):e0257297. doi: 10.1371/journal.pone.0257297 (PMC8496788; doi:10.1371/journal.pone.0257297)
Supplement: S1 Dataset — (DOCX) [file pone.0257297.s005.docx]

**Interviews coding matrix**

| **Code** | | | **Patient-associated aspects believed to influence the management of patients of non-Swedish origin** | | | **Students’ reactions and action associated with the management of patients of non-Swedish origin** | | |
| --- | --- | --- | --- | --- | --- | --- | --- | --- |
| **Informant** | **Case** | **Meaning unit** | Background of significance | Potential differences in medical culture | Obstacles to communication between patient and health care | Medical management | Problem solving | Students’ feelings and previous experience of influence |
| **E1** | 1 | 1 |  |  |  |  | 1 |  |
|  |  | 2 |  | 1 | 1 |  | 1 |  |
|  |  | 3 |  | 1 | 1 |  |  |  |
|  |  | 4 |  | 1 |  |  | 1 |  |
|  |  | 5 |  |  |  | 1 | 1 |  |
|  |  | 6 |  |  |  |  | 1 |  |
|  |  | 7 |  | 1 |  |  | 1 |  |
|  |  | 8 |  |  |  | 1 |  |  |
|  |  | 9 |  |  |  | 1 |  |  |
|  |  | 10 |  | 1 |  |  |  |  |
|  |  | 11 |  |  |  | 1 |  | 1 |
|  |  | 12 |  | 1 |  |  |  |  |
|  |  | 13 |  |  | 1 |  |  |  |
|  |  | 14 |  | 1 |  |  |  |  |
|  |  | 15 |  |  |  | 1 |  |  |
|  |  | 16 |  | 1 |  |  |  |  |
|  |  | 17 |  |  |  |  |  | 1 |
|  |  | 18 |  |  |  |  | 1 | 1 |
|  | 2 | 19 |  |  |  | 1 |  |  |
|  |  | 20 |  |  |  | 1 | 1 |  |
|  |  | 21 |  |  |  | 1 | 1 |  |
|  |  | 22 |  |  |  |  | 1 |  |
|  |  | 23 |  |  | 1 |  |  |  |
|  |  | 24 | 1 |  |  | 1 | 1 |  |
|  |  | 25 |  |  |  |  | 1 | 1 |
|  |  | 26 |  |  |  | 1 | 1 |  |
|  |  | 27 |  |  |  | 1 |  |  |
|  |  | 28 |  |  |  |  |  | 1 |
|  |  | 29 | 1 |  |  |  |  |  |
|  |  | 30 | 1 |  |  | 1 |  | 1 |
|  |  | 31 |  |  |  | 1 |  |  |
|  |  | 32 |  |  |  |  |  | 1 |
|  |  | 33 |  |  |  |  | 1 |  |
|  |  | 34 |  |  |  |  | 1 | 1 |
|  |  | 35 |  |  |  |  |  | 1 |
|  |  | 36 |  |  |  | 1 |  | 1 |
| **E2** | 1 | 1 | 1 |  |  | 1 |  |  |
|  |  | 2 |  | 1 |  |  |  |  |
|  |  | 3 | 1 |  |  | 1 |  |  |
|  |  | 4 |  | 1 | 1 |  |  |  |
|  |  | 5 |  |  | 1 | 1 | 1 |  |
|  |  | 6 |  | 1 |  |  | 1 |  |
|  |  | 7 |  | 1 |  |  |  | 1 |
|  |  | 8 |  |  | 1 |  | 1 | 1 |
|  |  | 9 |  |  |  |  | 1 | 1 |
|  |  | 10 |  |  |  | 1 |  |  |
|  |  | 11 |  |  | 1 |  | 1 |  |
|  |  | 12 |  | 1 |  |  |  |  |
|  |  | 13 |  | 1 |  |  |  |  |
|  |  | 14 |  |  |  | 1 | 1 | 1 |
|  |  | 15 |  |  |  | 1 | 1 | 1 |
|  | 2 | 16 |  |  |  | 1 |  |  |
|  |  | 17 |  |  |  | 1 |  |  |
|  |  | 18 |  |  |  | 1 | 1 |  |
|  |  | 19 | 1 | 1 |  |  | 1 |  |
|  |  | 20 |  | 1 |  |  |  |  |
|  |  | 21 | 1 |  |  |  |  |  |
|  |  | 22 |  |  |  | 1 |  | 1 |
|  |  | 23 |  |  |  | 1 | 1 | 1 |
|  |  | 24 |  |  |  |  |  | 1 |
| **E3** | 1 | 1 |  |  |  | 1 |  |  |
|  |  | 2 |  | 1 | 1 |  |  |  |
|  |  | 3 |  |  |  | 1 |  |  |
|  |  | 4 |  |  |  | 1 |  |  |
|  |  | 5 |  |  |  |  | 1 |  |
|  |  | 6 |  |  |  | 1 | 1 | 1 |
|  |  | 7 |  | 1 |  |  |  |  |
|  |  | 8 |  |  |  |  |  | 1 |
|  |  | 9 |  | 1 |  |  |  |  |
|  |  | 10 |  |  |  | 1 |  | 1 |
|  |  | 11 |  |  |  |  |  | 1 |
|  |  | 12 |  |  |  | 1 |  |  |
|  |  | 13 |  | 1 |  |  |  |  |
|  |  | 14 |  |  | 1 |  |  |  |
|  |  | 15 |  |  |  |  | 1 | 1 |
|  |  | 16 |  |  |  |  |  | 1 |
|  | 2 | 17 |  | 1 |  | 1 |  |  |
|  |  | 18 | 1 |  |  | 1 |  |  |
|  |  | 19 |  |  |  | 1 |  |  |
|  |  | 20 | 1 |  |  |  |  |  |
|  |  | 21 |  |  |  | 1 |  |  |
|  |  | 22 | 1 |  |  | 1 |  |  |
|  |  | 23 |  |  | 1 | 1 |  |  |
|  |  | 24 |  |  |  |  |  | 1 |
|  |  | 25 |  |  |  |  | 1 | 1 |
|  |  | 26 |  |  |  | 1 | 1 | 1 |
|  |  | 27 |  |  |  | 1 |  | 1 |
|  |  | 28 |  | 1 |  |  |  |  |
|  |  | 29 |  |  |  |  |  | 1 |
|  |  | 30 |  |  |  |  |  | 1 |
| **E4** | 1 | 1 |  |  |  | 1 |  |  |
|  |  | 2 |  | 1 |  |  |  |  |
|  |  | 3 |  | 1 | 1 |  |  |  |
|  |  | 4 |  |  |  | 1 | 1 |  |
|  |  | 5 |  |  |  |  | 1 |  |
|  |  | 6 |  |  |  |  | 1 |  |
|  |  | 7 |  |  | 1 |  | 1 | 1 |
|  |  | 8 |  | 1 |  |  | 1 |  |
|  |  | 9 |  | 1 |  |  |  |  |
|  |  | 10 |  |  |  | 1 |  |  |
|  |  | 11 |  | 1 |  |  | 1 |  |
|  |  | 12 |  |  |  | 1 |  | 1 |
|  |  | 13 |  | 1 |  |  | 1 |  |
|  |  | 14 |  |  |  | 1 |  | 1 |
|  |  | 15 |  |  |  |  |  | 1 |
|  |  | 16 |  |  |  |  |  | 1 |
|  | 2 | 17 | 1 |  |  | 1 |  |  |
|  |  | 18 |  |  |  | 1 |  |  |
|  |  | 19 |  |  |  | 1 |  |  |
|  |  | 20 | 1 |  |  |  |  |  |
|  |  | 21 |  |  |  | 1 |  |  |
|  |  | 22 |  |  |  | 1 |  | 1 |
|  |  | 23 |  |  |  | 1 |  | 1 |
|  |  | 24 |  |  |  |  |  | 1 |
|  |  | 25 | 1 |  | 1 |  |  |  |
|  |  | 26 | 1 |  |  | 1 |  |  |
|  |  | 27 |  |  |  | 1 |  | 1 |
|  |  | 28 |  |  | 1 |  | 1 | 1 |
|  |  | 29 |  |  |  |  | 1 |  |
|  |  | 30 |  |  |  | 1 | 1 | 1 |
| **E5** | 1 | 1 |  |  |  | 1 |  |  |
|  |  | 2 |  | 1 | 1 |  | 1 |  |
|  |  | 3 |  |  |  | 1 |  |  |
|  |  | 4 |  |  |  |  | 1 |  |
|  |  | 5 |  | 1 |  |  |  | 1 |
|  |  | 6 |  |  |  | 1 |  |  |
|  |  | 7 |  | 1 |  |  |  | 1 |
|  |  | 8 |  |  |  |  |  | 1 |
|  |  | 9 |  | 1 |  |  |  | 1 |
|  |  | 10 |  |  |  |  |  | 1 |
|  |  | 11 |  |  |  |  |  | 1 |
|  |  | 12 |  |  |  |  |  | 1 |
|  | 2 | 13 |  |  |  | 1 |  |  |
|  |  | 14 |  |  |  | 1 |  |  |
|  |  | 15 |  |  |  |  | 1 |  |
|  |  | 16 | 1 |  |  |  | 1 |  |
|  |  | 17 | 1 |  |  | 1 |  | 1 |
|  |  | 18 |  |  |  | 1 |  | 1 |
|  |  | 19 |  |  |  | 1 |  |  |
|  |  | 20 |  |  |  |  | 1 |  |
|  |  | 21 |  |  |  | 1 |  | 1 |
|  |  | 22 |  |  |  | 1 |  |  |
|  |  | 23 |  |  |  |  |  | 1 |
|  |  | 24 |  |  |  | 1 | 1 |  |
|  |  | 25 |  | 1 |  |  |  |  |
|  |  | 26 |  |  |  |  |  | 1 |
|  |  | 27 |  |  |  | 1 |  | 1 |
|  |  | 28 |  |  |  |  |  | 1 |
| **E6** | 1 | 1 |  |  |  | 1 |  |  |
|  |  | 2 | 1 |  |  | 1 |  |  |
|  |  | 3 |  | 1 | 1 |  |  |  |
|  |  | 4 |  |  |  | 1 | 1 | 1 |
|  |  | 5 |  |  |  |  | 1 | 1 |
|  |  | 6 |  |  |  |  | 1 | 1 |
|  |  | 7 |  |  |  | 1 |  | 1 |
|  |  | 8 |  |  |  | 1 |  | 1 |
|  |  | 9 |  |  |  |  |  | 1 |
|  |  | 10 |  | 1 | 1 |  |  | 1 |
|  |  | 11 |  | 1 |  |  |  |  |
|  |  | 12 | 1 | 1 |  |  |  |  |
|  |  | 13 |  |  |  | 1 |  |  |
|  |  | 14 |  | 1 |  |  |  |  |
|  |  | 15 |  |  |  |  | 1 | 1 |
|  |  | 16 |  |  |  |  |  | 1 |
|  | 2 | 17 |  |  |  |  | 1 |  |
|  |  | 18 | 1 |  |  | 1 |  |  |
|  |  | 19 |  |  |  | 1 | 1 |  |
|  |  | 20 | 1 |  |  |  | 1 |  |
|  |  | 21 |  |  |  |  | 1 |  |
|  |  | 22 | 1 |  |  | 1 |  | 1 |
|  |  | 23 | 1 |  |  |  |  | 1 |
|  |  | 24 |  |  |  | 1 |  | 1 |
|  |  | 25 | 1 |  |  | 1 |  | 1 |
|  |  | 26 | 1 |  |  |  | 1 | 1 |
|  |  | 27 |  |  |  |  |  | 1 |
|  |  | 28 |  |  |  | 1 |  |  |
| **E7** | 1 | 1 |  | 1 |  | 1 |  |  |
|  |  | 2 |  | 1 |  |  |  |  |
|  |  | 3 |  |  |  | 1 | 1 |  |
|  |  | 4 |  |  |  |  | 1 |  |
|  |  | 5 |  |  |  | 1 | 1 |  |
|  |  | 6 |  |  | 1 | 1 |  |  |
|  |  | 7 |  | 1 |  |  | 1 | 1 |
|  |  | 8 |  |  | 1 |  |  | 1 |
|  |  | 9 |  | 1 |  |  |  |  |
|  |  | 10 |  | 1 |  |  |  |  |
|  |  | 11 |  |  |  |  |  | 1 |
|  |  | 12 |  |  |  |  |  | 1 |
|  | 2 | 13 | 1 |  |  |  | 1 |  |
|  |  | 14 | 1 |  |  | 1 |  |  |
|  |  | 15 |  |  |  | 1 |  |  |
|  |  | 16 | 1 |  |  |  |  |  |
|  |  | 17 | 1 |  |  |  |  |  |
|  |  | 18 |  |  |  | 1 |  |  |
|  |  | 19 |  |  |  |  | 1 | 1 |
|  |  | 20 |  |  |  |  |  | 1 |
|  |  | 21 |  |  |  | 1 |  | 1 |
|  |  | 22 | 1 |  |  | 1 |  | 1 |
|  |  | 23 |  |  |  |  |  | 1 |
|  |  | 24 |  |  |  | 1 |  |  |
|  |  | 25 |  |  |  |  |  | 1 |
|  |  | 26 |  |  |  |  |  | 1 |
| **E8** | 1 | 1 |  |  |  | 1 |  |  |
|  |  | 2 |  |  | 1 |  | 1 |  |
|  |  | 3 |  |  |  | 1 | 1 |  |
|  |  | 4 |  |  |  |  | 1 |  |
|  |  | 5 |  |  |  | 1 | 1 | 1 |
|  |  | 6 |  |  |  |  |  | 1 |
|  |  | 7 |  | 1 |  |  | 1 |  |
|  |  | 8 |  |  |  | 1 |  |  |
|  |  | 9 |  |  | 1 |  | 1 |  |
|  |  | 10 |  | 1 |  |  |  |  |
|  |  | 11 |  |  |  |  | 1 | 1 |
|  |  | 12 |  | 1 | 1 |  | 1 |  |
|  |  | 13 |  |  |  |  | 1 | 1 |
|  | 2 | 14 |  |  |  | 1 |  |  |
|  |  | 15 |  |  |  | 1 |  |  |
|  |  | 16 |  |  |  | 1 | 1 |  |
|  |  | 17 | 1 |  |  | 1 |  |  |
|  |  | 18 | 1 |  |  | 1 |  |  |
|  |  | 19 | 1 |  |  | 1 |  | 1 |
|  |  | 20 |  | 1 |  | 1 | 1 |  |
|  |  | 21 |  |  |  |  | 1 |  |
|  |  | 22 |  |  |  | 1 |  | 1 |
|  |  | 23 |  |  |  | 1 | 1 |  |
|  |  | 24 | 1 |  |  |  | 1 |  |
|  |  | 25 |  |  |  | 1 |  | 1 |
|  |  | 26 |  | 1 | 1 |  |  | 1 |
|  |  | 27 |  |  |  | 1 |  | 1 |
|  |  | 28 |  |  |  | 1 |  |  |
|  |  | 29 |  |  |  |  | 1 | 1 |
|  |  | 30 | 1 |  |  |  |  | 1 |
| **E9** | 1 | 1 | 1 |  |  | 1 |  |  |
|  |  | 2 |  | 1 | 1 |  |  |  |
|  |  | 3 |  |  |  | 1 | 1 |  |
|  |  | 4 |  |  |  |  | 1 |  |
|  |  | 5 |  | 1 |  |  |  | 1 |
|  |  | 6 |  |  |  |  |  | 1 |
|  |  | 7 |  | 1 |  |  |  | 1 |
|  |  | 8 |  |  |  | 1 |  | 1 |
|  |  | 9 |  | 1 |  |  |  |  |
|  |  | 10 |  |  |  | 1 |  | 1 |
|  |  | 11 |  |  |  |  |  | 1 |
|  | 2 | 12 |  |  |  | 1 |  |  |
|  |  | 13 | 1 |  |  | 1 |  |  |
|  |  | 14 |  |  |  | 1 |  |  |
|  |  | 15 |  |  |  | 1 |  |  |
|  |  | 16 | 1 |  |  |  |  |  |
|  |  | 17 | 1 |  |  | 1 | 1 |  |
|  |  | 18 |  |  |  |  |  | 1 |
|  |  | 19 | 1 |  |  | 1 |  | 1 |
|  |  | 20 |  |  |  | 1 |  | 1 |
|  |  | 21 |  |  |  | 1 |  |  |
|  |  | 22 | 1 |  |  |  |  |  |
|  |  | 23 |  |  |  |  |  | 1 |
|  |  | 24 |  | 1 |  |  |  | 1 |
| **E10** | 1 | 1 |  |  |  | 1 |  |  |
|  |  | 2 |  |  | 1 |  | 1 |  |
|  |  | 3 |  |  |  |  | 1 |  |
|  |  | 4 |  |  |  |  | 1 |  |
|  |  | 5 |  | 1 |  |  | 1 | 1 |
|  |  | 6 |  | 1 | 1 |  |  | 1 |
|  |  | 7 |  | 1 |  |  |  |  |
|  |  | 8 | 1 | 1 | 1 | 1 |  |  |
|  |  | 9 |  |  |  | 1 |  |  |
|  |  | 10 |  | 1 |  |  |  | 1 |
|  |  | 11 |  |  |  |  | 1 |  |
|  |  | 12 |  | 1 |  |  |  |  |
|  |  | 13 |  |  |  |  | 1 | 1 |
|  |  | 14 |  |  |  |  | 1 | 1 |
|  |  | 15 |  |  |  |  |  | 1 |
|  | 2 | 16 |  |  |  | 1 | 1 |  |
|  |  | 17 |  |  |  | 1 | 1 |  |
|  |  | 18 | 1 |  |  |  | 1 |  |
|  |  | 19 | 1 |  |  |  |  |  |
|  |  | 20 |  |  |  | 1 |  |  |
|  |  | 21 |  |  |  |  | 1 |  |
|  |  | 22 |  |  |  | 1 |  |  |
|  |  | 23 |  | 1 |  |  |  |  |
|  |  | 24 |  | 1 |  |  |  |  |
|  |  | 25 |  |  |  |  | 1 |  |
|  |  | 26 |  |  |  | 1 |  |  |
|  |  | 27 |  |  |  |  | 1 | 1 |
|  |  | 28 |  |  |  | 1 |  | 1 |
|  |  | 29 |  |  |  |  |  | 1 |
|  |  | 30 | 1 |  |  | 1 |  | 1 |
|  |  | 31 |  |  |  | 1 |  | 1 |
|  |  | 32 | 1 |  |  |  |  |  |
|  |  | 33 | 1 |  |  |  |  | 1 |
|  |  | 34 |  |  |  |  |  | 1 |
|  |  | 35 |  |  |  | 1 |  | 1 |
| **E11** | 1 | 1 | 1 |  |  | 1 |  |  |
|  |  | 2 |  | 1 | 1 |  |  |  |
|  |  | 3 |  |  |  | 1 |  |  |
|  |  | 4 |  |  |  |  | 1 |  |
|  |  | 5 |  | 1 | 1 |  |  | 1 |
|  |  | 6 |  |  |  |  | 1 | 1 |
|  |  | 7 |  |  |  | 1 |  |  |
|  |  | 8 |  | 1 | 1 |  |  |  |
|  |  | 9 |  | 1 |  |  |  | 1 |
|  |  | 10 |  | 1 |  | 1 |  |  |
|  |  | 11 |  |  |  | 1 |  | 1 |
|  |  | 12 |  |  |  |  |  | 1 |
|  |  | 13 |  |  |  |  |  | 1 |
|  | 2 | 14 |  |  |  | 1 | 1 |  |
|  |  | 15 |  |  |  | 1 |  |  |
|  |  | 16 |  |  |  | 1 |  |  |
|  |  | 17 |  |  |  |  | 1 |  |
|  |  | 18 |  |  |  | 1 | 1 |  |
|  |  | 19 |  |  |  | 1 |  | 1 |
|  |  | 20 |  |  |  | 1 |  | 1 |
|  |  | 21 | 1 |  | 1 |  | 1 | 1 |
|  |  | 22 |  | 1 |  |  |  | 1 |
|  |  | 23 |  |  |  |  |  | 1 |
|  |  | 24 |  |  |  |  |  | 1 |
|  |  | 25 |  |  |  |  |  | 1 |
| **NE1** | 1 | 1 |  |  |  | 1 |  |  |
|  |  | 2 |  |  |  | 1 |  |  |
|  |  | 3 | 1 |  |  |  | 1 |  |
|  |  | 4 |  |  |  |  | 1 |  |
|  |  | 5 |  |  |  |  | 1 |  |
|  |  | 6 |  |  |  |  | 1 | 1 |
|  |  | 7 |  |  | 1 |  | 1 | 1 |
|  |  | 8 |  |  |  |  |  | 1 |
|  |  | 9 |  | 1 | 1 | 1 | 1 |  |
|  |  | 10 |  |  |  | 1 |  |  |
|  |  | 11 |  | 1 |  |  |  |  |
|  |  | 12 |  | 1 |  |  |  |  |
|  |  | 13 |  | 1 |  |  | 1 | 1 |
|  |  | 14 |  |  |  |  |  | 1 |
|  | 2 | 15 | 1 |  |  | 1 |  |  |
|  |  | 16 |  |  |  | 1 |  |  |
|  |  | 17 |  |  |  | 1 |  |  |
|  |  | 18 |  | 1 |  |  |  |  |
|  |  | 19 | 1 |  |  | 1 |  |  |
|  |  | 20 |  |  |  | 1 | 1 |  |
|  |  | 21 |  | 1 |  |  |  | 1 |
|  |  | 22 |  |  | 1 |  |  | 1 |
|  |  | 23 |  | 1 |  |  |  | 1 |
|  |  | 24 |  |  |  | 1 |  | 1 |
|  |  | 25 |  |  |  |  | 1 |  |
|  |  | 26 |  |  |  | 1 |  |  |
|  |  | 27 | 1 |  |  |  |  | 1 |
|  |  | 28 | 1 | 1 |  |  |  |  |
|  |  | 29 |  |  |  | 1 |  |  |
|  |  | 30 |  | 1 |  |  |  | 1 |
|  |  | 31 |  |  |  |  |  | 1 |
|  |  | 32 |  |  |  | 1 |  | 1 |
| **NE2** | 1 | 1 |  |  |  | 1 |  |  |
|  |  | 2 |  | 1 | 1 |  |  |  |
|  |  | 3 |  |  |  | 1 |  |  |
|  |  | 4 |  |  |  |  | 1 |  |
|  |  | 5 |  |  |  |  | 1 | 1 |
|  |  | 6 |  | 1 | 1 |  | 1 | 1 |
|  |  | 7 |  |  |  |  |  | 1 |
|  |  | 8 |  |  |  | 1 |  | 1 |
|  |  | 9 |  | 1 |  | 1 |  | 1 |
|  |  | 10 |  | 1 |  |  |  | 1 |
|  |  | 11 |  |  |  | 1 |  |  |
|  |  | 12 |  |  | 1 |  |  | 1 |
|  |  | 13 |  | 1 |  |  |  |  |
|  |  | 14 |  |  |  |  | 1 | 1 |
|  |  | 15 |  |  | 1 |  | 1 | 1 |
|  | 2 | 16 |  |  |  | 1 | 1 |  |
|  |  | 17 |  |  |  | 1 |  |  |
|  |  | 18 |  |  |  | 1 | 1 |  |
|  |  | 19 |  | 1 |  | 1 |  |  |
|  |  | 20 | 1 |  |  |  |  |  |
|  |  | 21 |  |  |  | 1 |  | 1 |
|  |  | 22 |  |  |  | 1 |  | 1 |
|  |  | 23 |  |  |  |  |  | 1 |
|  |  | 24 | 1 |  |  |  |  | 1 |
|  |  | 25 |  |  |  |  |  | 1 |
|  |  | 26 |  |  |  |  |  | 1 |
|  |  | 27 |  | 1 |  |  |  |  |
|  |  | 28 |  |  |  |  |  | 1 |
|  |  | 29 |  |  |  | 1 |  | 1 |
|  |  | 30 |  |  |  |  |  | 1 |
| **NE3** | 1 | 1 |  |  |  | 1 |  |  |
|  |  | 2 |  | 1 |  |  | 1 |  |
|  |  | 3 |  |  |  | 1 | 1 |  |
|  |  | 4 |  |  |  |  | 1 |  |
|  |  | 5 |  | 1 |  |  |  | 1 |
|  |  | 6 |  |  |  |  |  | 1 |
|  |  | 7 |  |  | 1 | 1 |  | 1 |
|  |  | 8 | 1 |  |  | 1 |  | 1 |
|  |  | 9 |  |  |  | 1 |  |  |
|  |  | 10 |  | 1 | 1 |  |  |  |
|  |  | 11 |  |  |  | 1 |  | 1 |
|  |  | 12 |  | 1 |  |  |  |  |
|  |  | 13 |  |  |  | 1 |  | 1 |
|  |  | 14 |  |  | 1 |  | 1 |  |
|  |  | 15 |  |  |  |  | 1 |  |
|  | 2 | 16 |  |  |  | 1 |  | 1 |
|  |  | 17 |  |  |  | 1 |  | 1 |
|  |  | 18 |  |  |  | 1 | 1 |  |
|  |  | 19 | 1 |  |  | 1 | 1 |  |
|  |  | 20 |  |  |  | 1 |  | 1 |
|  |  | 21 | 1 |  |  |  | 1 | 1 |
|  |  | 22 |  |  |  |  |  | 1 |
|  |  | 23 | 1 |  |  | 1 |  | 1 |
|  |  | 24 |  |  |  | 1 |  | 1 |
|  |  | 25 | 1 |  |  | 1 |  |  |
|  |  | 26 |  |  |  | 1 | 1 | 1 |
|  |  | 27 |  |  |  | 1 | 1 |  |
|  |  | 28 |  |  |  |  |  | 1 |
| **NE4** | 1 | 1 |  |  |  | 1 |  |  |
|  |  | 2 |  |  | 1 |  |  |  |
|  |  | 3 |  |  |  | 1 | 1 |  |
|  |  | 4 |  |  |  |  | 1 |  |
|  |  | 5 |  | 1 |  |  | 1 | 1 |
|  |  | 6 |  |  | 1 |  | 1 |  |
|  |  | 7 |  |  | 1 |  |  | 1 |
|  |  | 8 |  |  |  |  |  | 1 |
|  |  | 9 |  |  |  |  |  | 1 |
|  |  | 10 |  | 1 | 1 |  |  |  |
|  |  | 11 |  |  |  | 1 |  |  |
|  |  | 12 |  | 1 |  |  |  |  |
|  |  | 13 |  |  |  |  |  | 1 |
|  |  | 14 |  | 1 |  |  |  | 1 |
|  |  | 15 |  |  |  | 1 |  |  |
|  | 2 | 16 |  |  |  | 1 |  |  |
|  |  | 17 |  |  |  | 1 | 1 |  |
|  |  | 18 | 1 |  |  |  | 1 |  |
|  |  | 19 | 1 |  |  |  |  |  |
|  |  | 20 |  |  |  | 1 |  | 1 |
|  |  | 21 |  | 1 |  | 1 |  | 1 |
|  |  | 22 |  |  |  |  |  | 1 |
|  |  | 23 | 1 | 1 |  |  |  |  |
|  |  | 24 |  |  |  | 1 |  |  |
|  |  | 25 |  |  |  |  |  | 1 |
|  |  | 26 |  |  |  | 1 | 1 | 1 |
|  |  | 27 |  |  |  | 1 | 1 | 1 |
| **NE5** | 1 | 1 |  |  |  | 1 |  |  |
|  |  | 2 |  | 1 | 1 |  |  |  |
|  |  | 3 |  |  |  | 1 | 1 |  |
|  |  | 4 |  |  |  |  | 1 |  |
|  |  | 5 |  | 1 |  |  | 1 | 1 |
|  |  | 6 |  |  |  |  |  | 1 |
|  |  | 7 |  | 1 |  |  |  | 1 |
|  |  | 8 |  |  |  |  |  | 1 |
|  |  | 9 | 1 | 1 |  |  |  | 1 |
|  |  | 10 |  |  |  | 1 |  |  |
|  |  | 11 |  | 1 |  |  |  |  |
|  |  | 12 |  |  |  | 1 |  | 1 |
|  |  | 13 |  |  |  |  | 1 |  |
|  | 2 | 14 |  |  |  |  | 1 |  |
|  |  | 15 | 1 |  |  | 1 |  |  |
|  |  | 16 |  |  |  | 1 | 1 |  |
|  |  | 17 |  |  |  | 1 | 1 |  |
|  |  | 18 |  |  |  | 1 |  |  |
|  |  | 19 | 1 |  | 1 |  |  |  |
|  |  | 20 |  |  |  |  |  | 1 |
|  |  | 21 | 1 |  |  |  |  | 1 |
|  |  | 22 | 1 |  |  |  |  |  |
|  |  | 23 |  |  |  | 1 |  | 1 |
|  |  | 24 |  |  | 1 |  |  | 1 |
|  |  | 25 |  |  |  |  |  | 1 |
|  |  | 26 |  |  |  |  |  | 1 |
| **NE6** | 1 | 1 |  |  |  | 1 |  |  |
|  |  | 2 |  |  | 1 |  |  |  |
|  |  | 3 |  |  |  | 1 |  |  |
|  |  | 4 |  |  |  |  | 1 |  |
|  |  | 5 |  | 1 |  |  |  | 1 |
|  |  | 6 |  | 1 | 1 | 1 | 1 | 1 |
|  |  | 7 |  |  |  | 1 |  | 1 |
|  |  | 8 |  |  |  |  | 1 |  |
|  |  | 9 |  |  |  | 1 |  |  |
|  |  | 10 |  | 1 |  |  | 1 |  |
|  |  | 11 |  | 1 |  |  |  |  |
|  |  | 12 |  |  |  |  |  | 1 |
|  |  | 13 |  |  |  |  | 1 | 1 |
|  | 2 | 14 |  |  |  | 1 |  |  |
|  |  | 15 |  |  |  | 1 |  |  |
|  |  | 16 |  |  |  | 1 |  |  |
|  |  | 17 | 1 |  |  | 1 |  |  |
|  |  | 18 | 1 |  |  |  | 1 |  |
|  |  | 19 |  |  |  | 1 | 1 | 1 |
|  |  | 20 |  |  |  |  | 1 |  |
|  |  | 21 | 1 |  |  |  | 1 | 1 |
|  |  | 22 |  |  |  | 1 |  |  |
|  |  | 23 | 1 |  |  |  |  |  |
|  |  | 24 |  |  |  |  |  | 1 |
|  |  | 25 |  |  |  |  |  | 1 |
| **NE7** | 1 | 1 |  |  |  | 1 |  |  |
|  |  | 2 |  | 1 | 1 |  |  |  |
|  |  | 3 |  |  |  |  | 1 |  |
|  |  | 4 |  |  |  | 1 |  |  |
|  |  | 5 |  | 1 |  |  | 1 | 1 |
|  |  | 6 |  |  | 1 |  | 1 |  |
|  |  | 7 |  |  |  |  |  | 1 |
|  |  | 8 |  |  | 1 | 1 |  | 1 |
|  |  | 9 |  |  |  | 1 |  |  |
|  |  | 10 |  | 1 |  |  |  |  |
|  |  | 11 |  |  |  |  |  | 1 |
|  |  | 12 |  |  |  |  | 1 | 1 |
|  | 2 | 13 |  |  |  | 1 | 1 |  |
|  |  | 14 |  |  |  | 1 |  |  |
|  |  | 15 |  |  |  | 1 |  |  |
|  |  | 16 |  |  |  | 1 |  | 1 |
|  |  | 17 |  |  |  | 1 | 1 |  |
|  |  | 18 | 1 |  |  |  | 1 | 1 |
|  |  | 19 |  |  |  | 1 |  | 1 |
|  |  | 20 |  |  |  |  |  | 1 |
|  |  | 21 |  |  |  | 1 |  | 1 |
|  |  | 22 | 1 |  |  | 1 |  | 1 |
|  |  | 23 |  |  |  | 1 | 1 |  |
|  |  | 24 | 1 |  |  |  |  | 1 |
|  |  | 25 |  |  |  |  |  | 1 |
|  |  | 26 |  |  |  |  |  | 1 |
