# Supplementary material for: Global surgery for medical students – is it meaningful? A mixed-method study
Source: PLoS One. 2021 Oct 7;16(10):e0257297. doi: 10.1371/journal.pone.0257297 (PMC8496788; doi:10.1371/journal.pone.0257297)
Supplement: Attachment — Submitted filename: Response to Reviewers.docx [file pone.0257297.s007.docx]

Dear editor,

Thank you for considering our manuscript for publication. We also thank the reviewers for their thorough revision and recommendations the first and second time. We have updated the manuscript according to their comments and provide responses below.

**Review Comments to the Author**

Please use the space provided to explain your answers to the questions above. You may also include additional comments for the author, including concerns about dual publication, research ethics, or publication ethics. (Please upload your review as an attachment if it exceeds 20,000 characters)

**Reviewer #1:** The authors have adequately addressed most editor and reviewer comments. Other than reconsidering their limitations section (which currently only contains strengths, yet no study is flawless), I have no further comments and applaud the authors for their work.

**Response:**

We thank you for this remark and opportunity to reflect on our work. We have updated the strengths and limitations section.

**Reviewer #2:** The authors have nicely dressed the reviewer comments. It still remains that this is not a terribly actionable paper, yet the criteria for publication in this journal have been met.

**Response:**

We are grateful for your comments and push towards a more generous article.

**Journal Requirements:**

Please review your reference list to ensure that it is complete and correct. If you have cited papers that have been retracted, please include the rationale for doing so in the manuscript text, or remove these references and replace them with relevant current references. Any changes to the reference list should be mentioned in the rebuttal letter that accompanies your revised manuscript. If you need to cite a retracted article, indicate the article’s retracted status in the References list and also include a citation and full reference for the retraction notice.

**Response:**

All articles are still in print and no changes were made to the reference list.
